# fMRI BOLD signals in the left angular gyrus and hippocampus are associated with memory precision

**DOI:** 10.1162/IMAG.a.977

**Published:** 2025-11-10

**Authors:** Mingzhu Hou, Paul F. Hill, Luke R. Pezanko, Ayse N. Z. Aktas, Arne D. Ekstrom, Michael D. Rugg

**Affiliations:** Center for Vital Longevity and School of Behavioral and Brain Sciences, The University of Texas at Dallas, Dallas, TX, United States; Department of Psychology, University of Arizona, Tucson, AZ, United States; Evelyn McKnight Brain Institute, University of Arizona, Tucson, AZ, United States

**Keywords:** episodic retrieval, reinstatement, pattern similarity analysis

## Abstract

It has been proposed that the neural correlates of successful memory retrieval can be dissociated from the correlates of retrieval precision (fidelity). The specific findings supporting this proposal are, however, inconsistent across different studies. Here, functional magnetic resonance imaging (fMRI) was employed to examine these neural correlates in the context of a test of memory for spatial location that minimized temporal overlap between mnemonic and visuomotor processing. During the encoding phase, participants studied different object images, each placed at a random location on an invisible circle. At test, studied and new images were presented to the participants. The requirement was to make a covert recognition memory judgment to each image and to attempt to recall its studied location, guessing if necessary. A cue signaling the requirement to make a location memory judgment was presented 4 s after image onset. Memory precision was estimated as the angular difference between the originally studied location and the location signaled by the participant. In an analysis that combined the data from the present study and a closely similar prior study, we replicated prior reports that during retrieval fMRI BOLD activity in the left angular gyrus (AG) and the hippocampus tracks memory precision on a trial-wise basis. Linear mixed-effects modeling indicated that the activity in the two regions explained independent sources of variability in these judgments. In addition, multivoxel pattern similarity analysis revealed an item-level reinstatement effect (as indexed by encoding-retrieval overlap) in the left AG that was restricted to items associated with high precision judgments. These findings suggest that the hippocampus and the left AG play non-redundant roles in the retrieval and behavioral expression of high precision episodic memories.

## 1 Introduction

Episodic memory refers to the mnemonic processes that support consciously accessible memory for personally experienced, unique events (Tulving, 1983). Findings from recent behavioral studies employing continuous memory metrics suggest that episodic memory retrieval can be dissociated into two components: retrieval success (the accessibility of the memory) and precision (the level of correspondence between the originally experienced event and the remembered event) (Gellersen et al., 2024; Harlow & Yonelinas, 2016; Korkki et al., 2020; Sutterer & Awh, 2016). As we discuss below, whether success and precision engage common or dissociable neural processes is unclear.

**Fig. 1.**
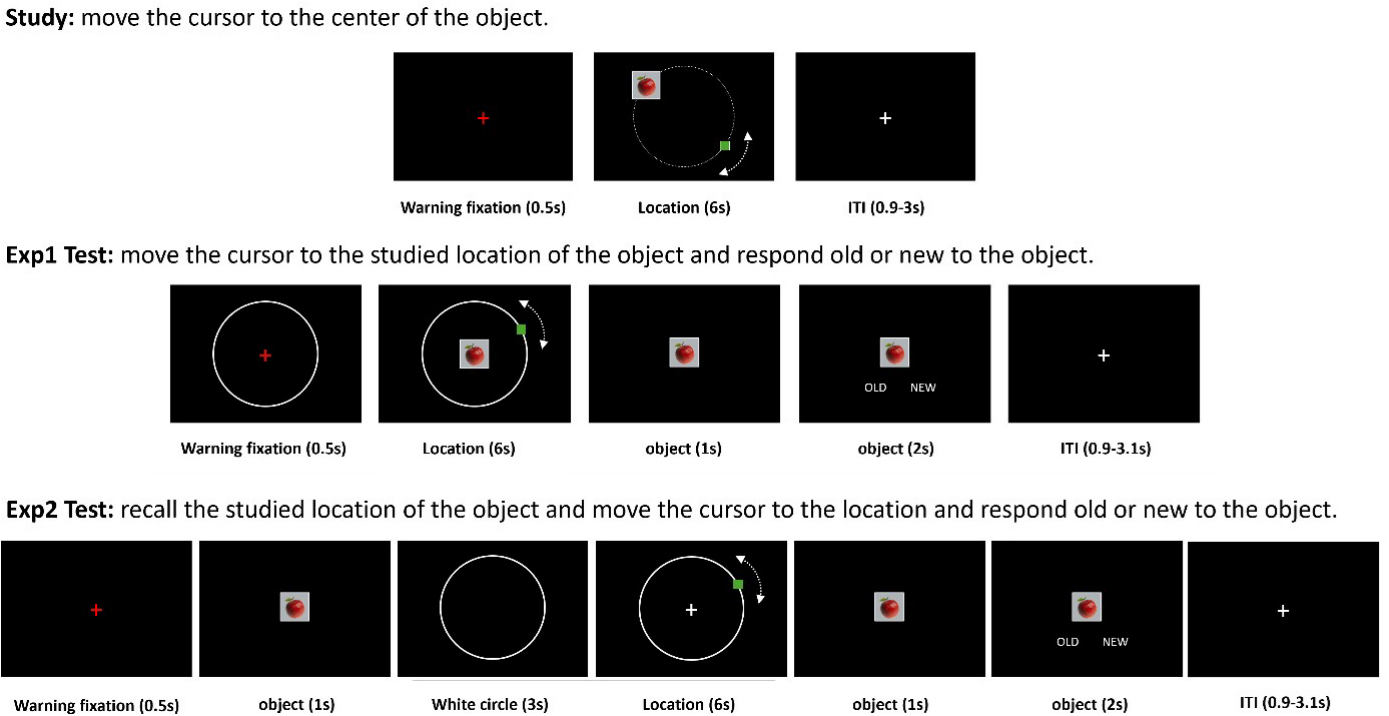
Schematic depiction of the study and test tasks for experiments 1 and 2. ITI: inter-trial interval.

Studies examining retrieval success and precision have typically employed ‘positional response accuracy’ tasks (Harlow & Donaldson, 2013). In these tasks, memory performance is assessed by a continuous metric that indexes the degree of correspondence between a feature of the study event and subsequent memory of that feature. For example, in our recent study (Hou et al., 2025) assessing memory precision for spatial location, participants studied a series of object images that were presented at different locations around a circle. In the subsequent test phase, both studied and new objects were individually presented at the center of the screen. Participants were instructed to manually control a cursor and move it until it overlapped with the studied location of the object, guessing if necessary (see Fig. 1: Exp1 Test). They then signaled whether the object was studied or unstudied. Thus, participant responses provided a continuous metric of memory accuracy (precision) in the form of distance error—the angular difference between the judged and the studied location of correctly recognized objects. The errors can be fit to a two-component mixture model comprising a rectangular distribution that reflects the probability of a guess, and a circular Gaussian distribution that allows estimation of the probability of successful retrieval with varying degrees of precision.

Using functional magnetic resonance imaging (fMRI), four studies of memory retrieval have examined the neural correlates of precision and success (Cooper et al., 2017; Hou et al., 2025; Korkki et al., 2023; Richter et al., 2016). Each study employed a positional response accuracy task and took a region of interest (ROI) approach to examine neural activity in the left angular gyrus (AG) and hippocampus. The results are mixed: Richter et al. (2016) and Cooper et al. (2017) reported that retrieval success was associated with enhancement of BOLD activity in the hippocampus, whereas memory precision was indexed by BOLD activity within the AG. By contrast, Korkki et al. (2023) reported that activity in both regions was sensitive to success and precision. In Hou et al. (2025), AG, but not hippocampal, activity scaled with precision, and we were unable to identify a neural correlate of success in either region. Additionally, Hou et al. identified an item-related reinstatement effect in the AG that was specific for memories retrieved with high precision. That is, there was a reliable overlap in the patterns of activity elicited in this region between the study and the test trials that included these items.

In addition to the ROI analyses described above, Hou et al. (2025) also compared test trials associated with different classes of memory judgment [successful location retrieval, guesses (unsuccessful location retrieval) and correct rejections of unstudied objects] at the whole brain level. Robust judgment effects were identified in a variety of cortical and subcortical regions, including regions that have previously been implicated in visuomotor function, such as the dorsal parietal cortex (DPC), premotor cortex, and the cerebellum. The effects primarily took the form of differences in the activity elicited by the correct rejections relative to the other trial types. However, the task design employed by Hou et al. (2025; see Fig. 1) meant that location recall and the associated location judgment temporally overlapped. This led us to conjecture that some of these effects might reflect the greater visuomotor demands associated with memory-guided responding than those associated with the requirement merely to randomly place a cursor somewhere on the test screen.

Here, we aimed to further investigate the neural correlates of retrieval success and precision. We employed an experimental design similar to that employed by Hou et al. (2025) but, crucially, we modified the test phase to incorporate a covert memory recall phase prior to the requirement to make the memory judgment, thereby temporally separating (and, we hoped, unconfounding) memory retrieval from the visuomotor demands of the associated judgment. Thus, we aimed to determine the extent to which the prior whole-brain findings reported by Hou et al. (2025) and described above were a consequence of the demands of the continuous performance task rather than reflections of mnemonic processing. Additionally, and as described below, we were also able to combine the data from the present and prior experiments to examine the neural correlates of precision and success with greater statistical power than that afforded by the individual experiments. To differentiate the two experiments, the experiment described by Hou et al. (2025) is referred to below as experiment 1, and the current study is referred to as experiment 2.

## 2 Methods

The methods employed in experiment 1 have been described in detail previously (Hou et al., 2025). Here, we focus on experiment 2 and briefly describe the differences between the experiments. As detailed below, to maximize the comparability of the estimated neural signals, we used the same boxcar duration to model the neural activity in each experiment. Equivalent definitions of location hits, as well as those for ‘high’ and ‘low’ precision trials were employed across experiments for the same reason. Note that the results were essentially the same when experiment-specific boxcar durations and precision-based definitions were employed.

### 2.1 Participants

Participants in experiment 2 comprised 24 young adults (mean age = 23 yrs, age range = 18-33 yrs, SD = 4.3, 11 female). A non-overlapping sample of 23 young adults participated in experiment 1. All participants were cognitively healthy, right-handed, had normal or corrected-to-normal vision, no history of neurological or psychiatric illness, and were not taking prescription medications that affected the central nervous system.

Informed consent was obtained in accordance with the University of Texas at Dallas Institutional Review Board guidelines. Participants were compensated at the rate of $30 an hour. For experiment 2, data from two additional participants were excluded due to near-chance levels of recognition memory.

### 2.2 Experimental items

Stimuli for both experiments comprised 136 images of everyday objects (see Fig. 1). 102 of the images were randomly employed as study items and the remaining 34 images were employed as new (unstudied) test items.

### 2.3 Procedure

The scanned study and test tasks employed in each experiment are schematized in Figure 1. As is evident from the figure, there was a single study block followed by two consecutive test blocks in experiment 1, and by four consecutive test blocks in experiment 2.

At study, each image was presented for 6 s at a random location on a virtual circle (radius = 4.9°). A small green cursor was also presented on the circle at least 60° distant from the image. Participants were instructed to use either the left or right button on a button box to move the cursor around the circle until it occupied the center of the image (see Fig. 1), and to remember both the image and its location.

At test, all studied images were intermixed with 34 new images. In experiment 1, each test block contained 51 studied images and 17 new images. In experiment 2, there were four test blocks in total: two blocks contained 26 studied images and 8 new images each, while the other two contained 25 studied images and 9 new images each.

The ordering of the test images within each block was pseudo-randomized so that no more than 3 old or new images occurred in succession. In experiment 2, the test image was presented for 1 s, followed by a 3-s presentation of a white circle with the same radius as the virtual circle employed at study. Participants were instructed to recall the studied location of the image and then to wait until a white fixation cross and a green cursor appeared on the display. They were then to use the button box to move the cursor (which was randomly placed on the circle) to the recalled location, guessing if necessary. After moving the cursor, participants signaled whether the presented object had been studied or not. As can be seen from Figure 1, the test procedure in experiment 1 differed, in that there was no delay between the presentation of the test image and the requirement to use the cursor to indicate its studied location.

The experimental display was viewed via a mirror mounted on the scanner head coil that reflected a back projected image (viewing distance 102 cm). Participants used the buttons under their right index and middle fingers to move the cursor in both the study and test phases and, at test, to respond ‘old’ or ‘new’ to the object image. The mappings between the button presses and the direction of the cursor movement, as well as the old/new judgments, were counterbalanced across participants in each experiment.

A 30-s break was given in the middle of each study and test block in experiment 1, as well as in the study block of experiment 2. During the break, a ‘rest’ cue was presented at the center of the screen. Participants were instructed to relax until the cue disappeared. In both experiments, the inter-trial interval varied randomly between 0.9 and 3 s during the study phase and between 0.9 and 3.1 s during the test phase.

### 2.4 MRI acquisition and preprocessing

MRI acquisition and preprocessing methods were identical across the two experiments. The data were acquired with a Siemens PRISMA 3T MR scanner equipped with a 32-channel head coil. Functional images were acquired from both the study and the test phases, with a T2^*^-weighted echoplanar sequence [TR = 1560 ms, TE = 30 ms, flip angle = 70°, field-of-view (FOV) = 220 mm, multiband factor = 2, 48 slices, voxel size = 2.5 x 2.5 x 2.5 mm, 0.5 mm inter-slice gap, anterior-to-posterior phase encoding direction]. A T1-weighted image was acquired with a Magnetization-Prepared Rapid Acquisition Gradient Echo pulse sequence (TR = 2300 ms, TE = 2.41 ms, FOV 256 mm, voxel size = 1 x 1 x 1 mm, 160 slices, sagittal acquisition). A field map was acquired after the functional scans using a double-echo gradient echo sequence (TE 1/2 = 4.92 ms/7.38 ms, TR = 520 ms, flip angle = 60°, FOV = 220 mm, 48 slices, 2.5 mm slice thickness). Eye movement data were collected from both the study and retrieval tasks in experiment 2. They will be reported in a separate publication.

Data preprocessing was performed with the SPM12 software package (Wellcome Department of Imaging Neuroscience, London, UK: www.fil.ion.ucl.ac.uk/spm) implemented in MATLAB R2018b (experiment 1) and MATLAB 2023a (experiment 2). The functional volumes were field-map corrected, realigned to the across-run mean image, reorientated to the anterior commissure–posterior commissure line, and spatially normalized to SPM’s MNI EPI template. The normalized images were resampled to 2.5 mm isotropic voxels and then smoothed with a 6 mm Gaussian kernel. Before they were entered into the participant-wise GLMs, the functional data from the different test blocks were concatenated using the spm_concatenate.m function. Anatomical images were normalized to SPM’s MNI T1 template.

### 2.5 Behavioral analyses

#### 2.5.1 Item recognition performance

Item memory (Pr) was calculated as the proportion of correctly recognized old items (item hits) minus the proportion of new items that were incorrectly endorsed as old (false alarms).

#### 2.5.2 Retrieval success and precision

Measures of retrieval success and precision were derived from the distributions of distance error (the angular difference between judged and actual study location in deg) of item hits. To enable comparability with prior findings, the distributions were fit to a two-component mixture model using the standard mixture modeling procedure implemented in the MemToolbox (Suchow et al., 2013). The model estimated a rectangular distribution reflecting the proportion of ‘guess’ responses (g), and a von Mises distribution that captured accurate location memory judgments of varying precision [characterized by the concentration parameter (Kappa) of the distribution]. Participant-wise g and Kappa metrics were estimated with the FitMultipleSubjects_MLE() function in MemToolbox. As in prior studies (e.g., Hou et al., 2025; Korkki et al., 2023; Richter et al., 2016), we estimated the retrieval success rate (pT) as 1 – g. In addition to Kappa, we also calculated a model-independent metric of memory precision, the mean absolute distance error of item hit trials for each participant (Gellersen et al., 2024; Nilakantan et al., 2018). Unlike Kappa, this metric is meaningful at the single-trial level and was employed in the trial-wise fMRI analyses reported below. For clarity, hereafter we refer to Kappa as the ‘model-derived metric of precision’, and absolute distance error as ‘precision’.

For each experiment, across-participant distance errors were fit to the mixture model using the MLE() function. Item hit trials were categorized as successful (location hit) and guess trials based on whether the associated distance errors had a <5% chance of fitting the group-wise von Mises distribution as estimated with the “vonmisecdf” function from www.paulbays.com/toolbox/.

### 2.6 fMRI analyses

#### 2.6.1 Regional trial type analyses

Using a whole brain analysis based on an F contrast among location hits, guesses, and correct rejections we identified robust effects of trial type in several cortical regions in our original report (Hou et al., 2025). As was mentioned in the introduction, the identified effects might reflect the varying visuomotor demands that were associated with the different trial types. In experiment 2, we sought to reduce these demands during memory retrieval by including a covert memory recall phase prior to the memory judgment phase (see Fig. 1). To examine whether this procedural modification influenced the whole-brain effects identified in Hou et al. (2025), we constructed participant-level GLMs analogous to those described in the original report. For the participant-wise GLMs, a 4 s boxcar function, which onset concurrently with the onset of the test item, was used to model neural activity synchronized to item onset in both experiments. Three event types of interest were included: location hits (item hits with absolute distance error <47°, the model-based cutoff for the location hit based on data from experiment 1), guesses (item hits with absolute distance error >46°), and correct rejections (CRs; correctly categorized unstudied items). For the sake of comparability with the whole brain analysis approach employed in Hou et al. (2025, see also Cooper et al., 2017; Korkki et al., 2023; Richter et al., 2016), a trial-wise measure of memory precision for location hits was included as a parametric modulator [although not reported here, as it is redundant with the linear mixed effects analyses reported below, the results derived from the parametric modulation model were highly similar to those reported in Hou et al. (2025)]. False alarms, misses, and trials with absent or multiple old/new responses were modeled as events of no interest. The GLMs also included as covariates six regressors modeling motion-related variance (three for rigid-body translation and three for rotation) and two (for experiment 1) or four (for experiment 2) constants for means across test blocks. Data from volumes with a transient displacement (relative to the prior volume) of >1 mm or >1° in any direction were modeled as covariates of no interest.

Parameter estimates were extracted from voxels within a 5 mm radius of the peak of each cluster identified in Hou et al. (2025) and subjected to a 3 (trial type) x 8 (region) x 2 (experiment) mixed-effects ANOVA. The MNI coordinates of each peak are listed in Table 1.

**Table 1.** Regions demonstrating significant trial type effects in the whole-brain analysis in Hou et al. (2025).

Region	MNI		
x	y	z
Left dorsal parietal cortex (DPC)	-26	-62	52
Right DPC	14	-72	52
Left premotor cortex	-23	-2	52
Right premotor cortex	30	3	55
Left dorsal prefrontal cortex (DLPFC)	-56	26	32
Left AG	-56	-70	32
Right AG	62	-52	38
Left cerebellum	-20	-84	-38

#### 2.6.2 Regional categorical precision analyses

We conducted targeted univariate and multi-voxel pattern similarity analyses to examine the neural correlates of retrieval success and precision, focusing on fMRI signals in the left AG and the hippocampus. We employed a combined region of interest (ROI) in the left AG based on the peak precision effects reported by Korkki et al. (2023) and Richter et al. (2016) (MNI coordinates: -54, -66, 33 and -54, -54, 33). The combined AG ROI comprised all voxels falling within a 5-mm radius of each peak, and is equivalent to the AG ROI employed by Hou et al. (2025). Note that results from this combined AG ROI were highly consistent with those obtained using the anatomically defined AG ROI described in section 2.6.6 (see Supplementary Materials). The anatomically defined left and right hippocampi served as the hippocampal ROIs. These ROIs took the form of bilateral hippocampal masks that had been manually traced on the group mean T1 images averaged across the two experiments.

As in Hou et al. (2025), we contrasted the BOLD activity elicited by item hit trials according to the level of memory precision. Three event types were modeled: high-precision hits (item hits with absolute distance error <16°), low-precision hits (item hits with absolute distance error ranging from 25–40°), and guesses (item hits with absolute distance error > 59°). Mean trial numbers for high-precision hits, low-precision hits, and guesses were 23 (range = 5–52), 11 (4–20), and 26 (5–43) for experiment 1, and 34 (13–61), 10 (2–17), and 20 (7–35) for experiment 2. Note that the results were unchanged after excluding data from participants with fewer than 5 low-precision hit trials. We employed an equivalent accuracy range (15° angular error) when defining high and low precision trials in each experiment to maintain consistency as to what constituted a ‘precise’ vs. an ‘imprecise’ location judgment. The 10-deg boundary between the high vs low-precision trials was inserted to amplify possible categorical differences between trial types. For the same reason, guess trials were defined as those associated with a distance error substantially below the cut-off. All other events were modeled as events of no interest.

Parameter estimates associated with the different trial types were extracted from the predefined AG and hippocampal ROIs. Parameter estimates extracted from the left and right hippocampus were averaged, given that preliminary analysis revealed no significant effects of hemisphere. AG and hippocampal parameter estimates were subjected to a repeated-measures ANOVA employing the factors of region (AG, hippocampus), trial type (high-precision, low-precision, guess), and experiment. Precision effects were operationalized as greater activity for high-precision than low-precision trials, while retrieval success effects were defined as greater activity for low-precision trials than guesses.

#### 2.6.3 Additional categorical precision analyses in the hippocampus

We conducted additional exploratory analyses to further examine the role of the hippocampus in memory precision and success. Participant-wise parameter estimates derived from the first-level GLMs described in the preceding section were subjected to mixed-effects ANOVA (factors of trial type and experiment) implemented in SPM12. To identify voxels where activity varied across the three trial types (high-precision hits, low-precision hits, guesses) in an unbiased manner, we employed an F contrast with thresholds of p < 0.05 and 20 contiguous voxels (see Bakker et al., 2008 and Lacy et al., 2011 for examples of a similar approach). The contrast, which leaves the profile of response magnitudes across the trial types free to vary, was restricted to voxels that fell within the bilateral hippocampal mask. Participant-wise mean across-voxel parameter estimates were extracted from each cluster identified with the contrast and entered into a 3 (trial type) x 2 (experiment) mixed ANOVA.

#### 2.6.4 Single-trial GLM

In each experiment, participant-wise single-trial beta estimates from both the study and test phases were derived from first-level GLMs employing the least squares all (LSA) approach (Abdulrahman & Henson, 2016; Mumford et al., 2012). Trial-wise neural activity elicited by the study items was modeled with a 6 s duration boxcar. Each study event was modeled as a separate event of interest. The 30 s rest period, the six motion regressors, and a constant modeling the mean BOLD signal in the block were included as covariates of no interest. An analogous single-trial GLM was constructed for the test blocks, with event-related neural activity modeled as a 4 s boxcar.

#### 2.6.5 Linear mixed-effects analyses

We constructed a set of linear mixed effects (LME) regression models to examine possible associations between trial-wise BOLD activity in the left AG and hippocampus during retrieval and the absolute distance error of location hits. An additional LME model was employed to ascertain whether any identified associations were independent of each other. We employed across-voxel mean parameter estimates derived from the AG ROI and the functionally defined hippocampal cluster (see section 2.6.3) as predictors of absolute distance error. The models were estimated in R (R core Team 2018) using the lmer function in the lme4 package (Bates et al., 2015). They followed the general format:

Absolute distance error = neural activity + exp + neural activity * exp + (1| participant)

where absolute distance error refers to the absolute distance between the estimated and actual studied location in deg (0-46°), neural activity refers to trial-wise BOLD signals in either left AG or the hippocampus, and exp refers to experiment (experiment 1 coded as 0, experiment 2 coded as 1). Participants were modeled as a random effect with a variable intercept. Because a singular fit error indicative of model overfitting was evident in most of the initial models, random slopes for the variable of neural activity were not specified.

#### 2.6.6 Multi-voxel pattern similarity analyses

We conducted multi-voxel pattern similarity analyses (PSA) to examine whether memory precision was associated with the level of correspondence between item-specific patterns of neural activity elicited during encoding and retrieval. Specifically, we examined retrieval-related reinstatement effects associated with high-precision location hits, low-precision location hits and guess trials. As in Hou et al. (2025), we employed three ROIs, namely, the left and right anatomically defined hippocampus and left angular gyrus [the ‘PGap’ parcel specified in the Anatomy Toolbox v3.0 (Eickhoff et al., 2005, 2006, 2007)]. Encoding- and retrieval-related trial-wise parameter estimates were extracted from all voxels falling within a given ROI. For any given item, reinstatement was operationalized as the difference between within-item and across-item study-test similarity (estimated as the Fisher-z transformed correlation). Within-item study-test similarity was calculated as the across-voxel correlation between a specific study trial and its corresponding test trial. Across-item similarity was computed as the mean of the correlations between the same study trial and all other test trials belonging to the same trial type. For each participant, the estimates of reinstatement were averaged across all trials within each trial class. Because no significant hemisphere effects were found in preliminary analyses, reinstatement estimates from the left and right hippocampus were averaged. Across-experiment one-sample t-tests were employed to assess whether the group-wise measures of reinstatement differed reliably from zero. For any ROI that demonstrated a reinstatement effect for at least one trial type, a trial type (high-precision, low-precision, guess) x experiment mixed-effects ANOVA was conducted to examine the possible relationship between reinstatement strength and memory precision.

Statistical analyses were conducted with SPSS 27.0 and R software (R Core Team, 2018). Nonsphericity between the levels of repeated-measures factors in the ANOVAs was corrected with the Greenhouse– Geisser procedure (Greenhouse & Geisser, 1959). Significance levels for follow-up pairwise contrasts were set at p < 0.05 after family-wise correction for multiple comparisons using the Bonferroni procedure. Results that failed to survive the correction are noted accordingly.

## 3 Results

### 3.1 Behavioral results

**Fig. 2.**
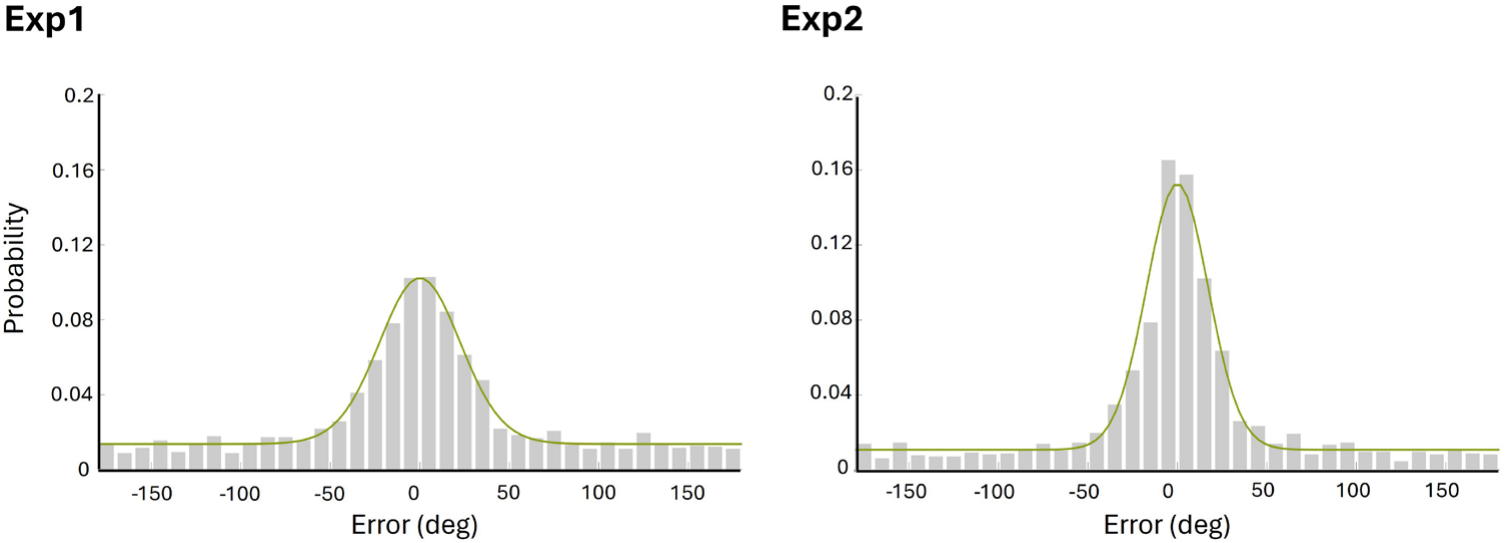
Distribution of distance errors for item hit trials across participants in experiment 1 and 2 (trial number = 1777 and 1917 for experiment 1 and 2, respectively). Each curve indicates the fit of the von Mises + uniform distributions mixture model.

The distributions of distance errors of all item hits pooled across participants from each experiment are illustrated in Figure 2. The model-derived estimate of precision (Kappa) was 6.43 for experiment 1 and 11.04 for experiment 2. The retrieval success rate (pT) was 0.51 and 0.62 for experiments 1 and 2, respectively. The model-based cutoff for the location hit vs guess trials was estimated as +/- 47° for experiment 1 and +/- 35° for experiment 2.

Table 2 shows participant-wise memory performance in each experiment. Both the mean Kappa and the absolute distance error for item hits differed significantly between experiment 1 and 2, indicative of higher precision in experiment 2. Item recognition performance was comparable between the two experiments.

**Table 2.** Mean participant-wise performance for the spatial precision task and item recognition.

	Exp 1	Exp 2	t	p
Kappa	5.38 (2.59)	12.43 (6.37)	5.01	<0.001
Guess rate	0.43 (0.20)	0.39 (0.17)	0.82	0.415
Absolute distance error	53.99 (15.39)	42.16 (12.72)	2.88	0.006
Item hit rate	0.76 (0.11)	0.78 (0.13)	0.74	0.465
Correct rejection rate	0.81 (0.13)	0.86 (0.12)	1.23	0.224
Pr	0.60 (0.19)	0.65 (0.17)	1.14	0.259

### 3.2 fMRI results

#### 3.2.1 Regional trial type analyses

**Fig. 3.**
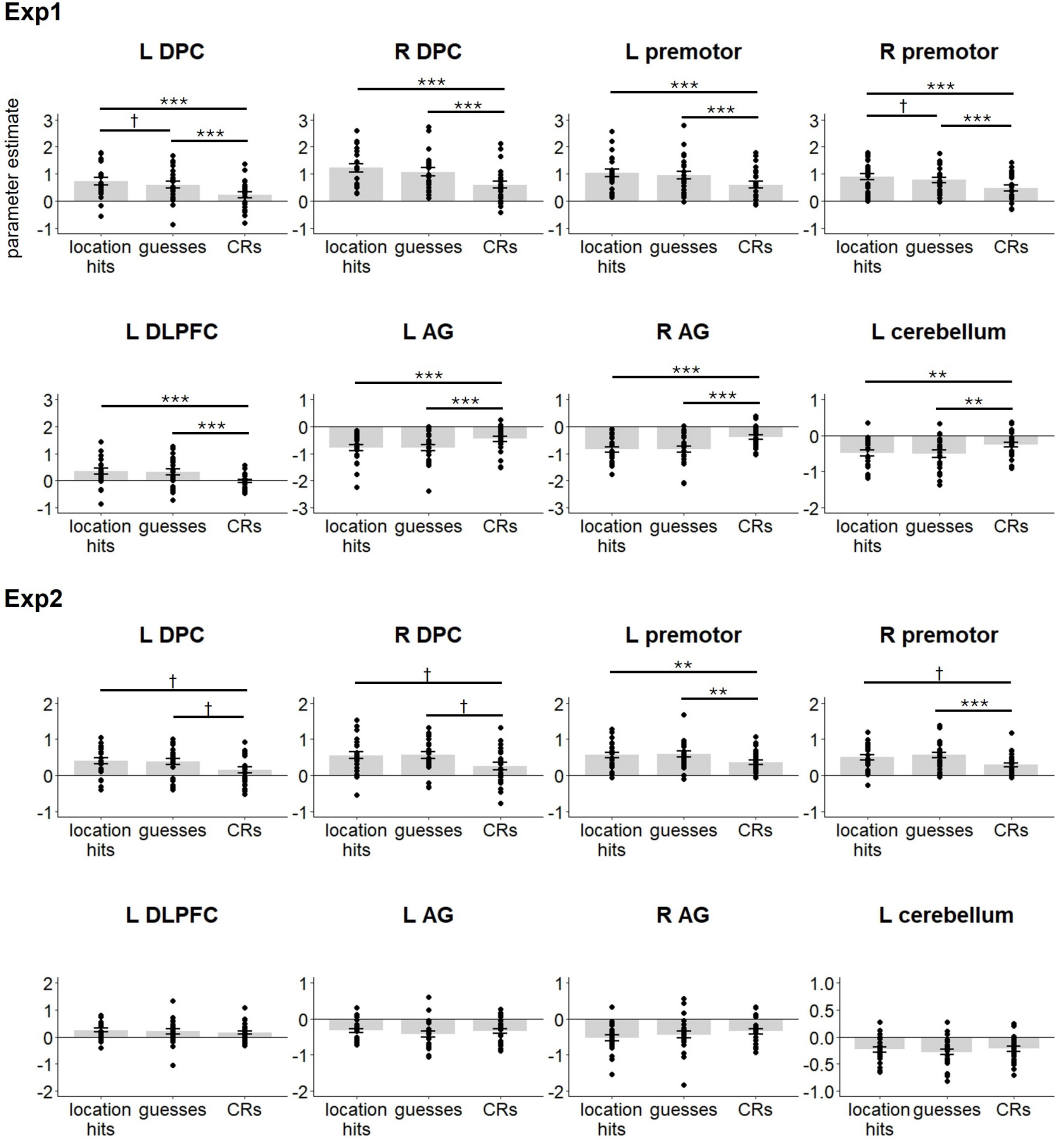
Parameter estimates extracted from the cortical regions identified by the whole brain analyses in Hou et al. (2025). Error bars indicate standard errors of the mean. **p < 0.01, ***p < 0.001, ^†^ non-significant after Bonferroni correction.

As described in section 2, we examined the effects of trial type in the regions originally identified in the whole-brain analysis of Hou et al. (2025). Results of the ANOVA contrasting the parameter estimates for location hits, guesses, and CRs across regions are shown in Table 3. As is evident from the table, there was a significant trial type x region x experiment interaction, indicating that the effect of trial type varied with both region and experiment. To unpack the interaction, we conducted pairwise t-tests among trial types in each region and experiment (24 pairwise contrasts for each experiment). As is illustrated in Figure 3, significant trial type differences were identified in all regions in experiment 1 (as expected, given that this is how these regions were identified). Analogous differences were identified in experiment 2 in bilateral DPC and premotor cortex, but no trial type differences were evident in this experiment in the remaining four regions. Note that the effects observed in the bilateral DPC, as well as the difference between location hits and CRs in the right premotor cortex in experiment 2 did not survive the Bonferroni correction.

**Table 3.** Results of ANOVAs comparing location hits, guesses, and CR trials in the regions identified by Hou et al. (2025).

	Results
Trial type	F(1.90, 85.66) = 7.75, p < 0.001, partial η^2^ = 0.147
Region	F(3.32, 149.33) = 92.99, p < 0.001, partial η^2^ = 0.674
Experiment	F(1, 45) = 1.72, p = 0.196, partial η^2^ = 0.037
Trial type x region	F(5.29, 237.97) = 27.26, p < 0.001, partial η^2^ = 0.377
Trial type x experiment	F(1.90, 85.66) = 0.46, p = 0.624, partial η^2^ = 0.010
Region x experiment	F(3.32, 149.33) = 7.33, p < 0.001, partial η^2^ = 0.140
Trial type x region x experiment	F(5.29, 237.97) = 6.10, p < 0.001, partial η^2^ = 0.119

#### 3.2.2 Regional categorical precision analyses

**Fig. 4.**
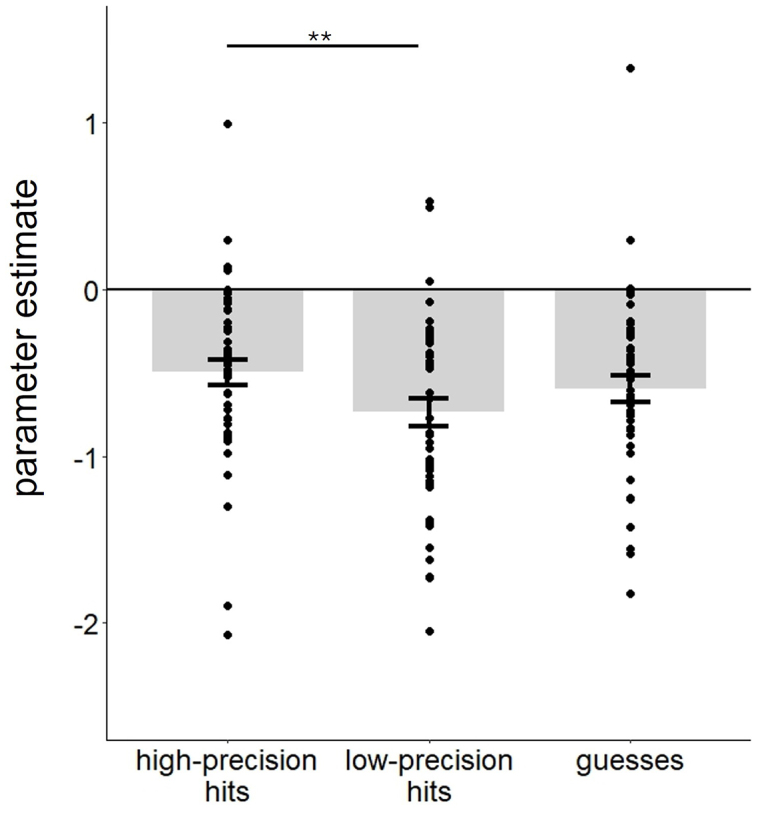
Parameter estimates of high-precision, low-precision, and guess trials from the AG ROI. Error bars indicate standard errors of the mean. **p < 0.01.

The results of the ANOVA contrasting the parameter estimates associated with high precision, low precision, and guess trials in the AG and hippocampal ROIs are listed in Table 4. As is shown in the table, the ANOVA gave rise to significant main effects of region, trial type, and experiment, as well as two-way interactions of region x experiment and region x trial type. The region x experiment interaction reflected the fact that while parameter estimates were more positive in experiment 2 than experiment 1 (p= 0.002 and 0.037 respectively), this difference was larger in the AG than the hippocampus (partial η^2^ = 0.19 and 0.09, respectively). Of importance, as evidenced by the non-significant three-way interaction, these between-experiment effects were additive with the effects of trial type. To unpack the region x trial type interaction, a separate one-way ANOVA (factor of trial type) was conducted for each ROI. In the AG, but not the hippocampus, the main effect of trial type was significant [F(1.99, 91.36) = 4.92, p = 0.009, partial η^2^ = 0.97, for the hippocampal ROI, p = 0.259]. Pairwise comparisons for the AG revealed that BOLD activity elicited by high-precision hits was greater than that for low-precision hits [t(46) = 3.01, p = 0.004, Cohen’s d = 0.44, see Fig. 4, while no other trial type contrasts were significant (ps > 0.077)].

**Table 4.** Results of the ANOVA comparing high-precision, low-precision, and guess trials in the a priori defined AG and the hippocampal ROIs.

	Results
Region	F(1, 45) = 79.77, p < 0.001, partial η^2^ = 0.64
Trial type	F(1.92, 86.44) = 4.26, p = 0.018, partial η^2^ = 0.09
Experiment	F(1, 45) = 11.66, p = 0.001, partial η^2^ = 0.21
Region x trial type	F(1.97, 86.44) = 3.44, p = 0.037, partial η^2^ = 0.07
Region x experiment	F(1, 45) = 6.82, p = 0.012, partial η^2^ = 0.13
Trial type x experiment	F(1.92, 86.44) = 0.15, p = 0.849, partial η^2^ < 0.01
Region x trial type x experiment	F(1.97, 88.65) = 0.14, p = 0.866, partial η^2^ < 0.01

#### 3.2.3 Additional categorical precision analyses in the hippocampus

**Fig. 5.**
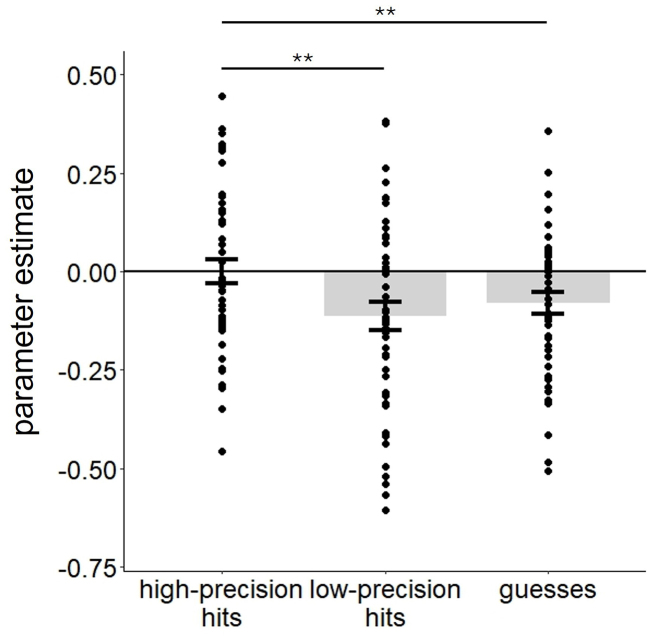
Parameter estimates associated with high-precision, low-precision and guess trials from the cluster identified by the ANOVA targeted on the hippocampus. Error bars indicate standard errors of the mean. **p < 0.01.

Employing the approach described in section 2.6.3, we identified a single cluster in the right hippocampus (peak 35, -20, -18, k = 41) that demonstrated a significant main effect of trial type. We conducted a 3 (trial type) x 2 (experiment) ANOVA on the parameter estimates extracted from the cluster, the results of which are shown in Table 5. Unsurprisingly, given how the cluster was identified, there was a main effect of trial type. Crucially, and undetermined by the main effect, follow-up pairwise t-tests indicated that the parameter estimates associated with high-precision trials were greater than those for either low-precision or guess trials [respectively, t(46) = 3.33, p = 0.002, Cohen’s d = 0.49; t(46) = 3.11, p = 0.003, Cohen’s d = 0.45]. The latter two trial types did not significantly differ (p = 0.293, see Fig. 5). Therefore, this analysis identified a significant precision effect in the right hippocampus. The main effect of experiment was also significant, reflecting greater activity in experiment 2 (M = - 0.01) than experiment 1 (M = -0.12).

**Table 5.** Results of ANOVAs comparing item hit trials associated with high, low precision and guess in the functionally defined hippocampal cluster.

	Results
Hippocampal cluster	
Trial type	F(1.82, 81.80) = 7.16, p = 0.002, partial η^2^ = 0.137
Experiment	F(1, 45) = 5.33, p = 0.026, partial η^2^ = 0.106
Trial type x experiment	F(1.82, 81.80) = 0.37, p = 0.669, partial η^2^ = 0.008

#### 3.2.4 Linear mixed-effects analyses

**Fig. 6:**
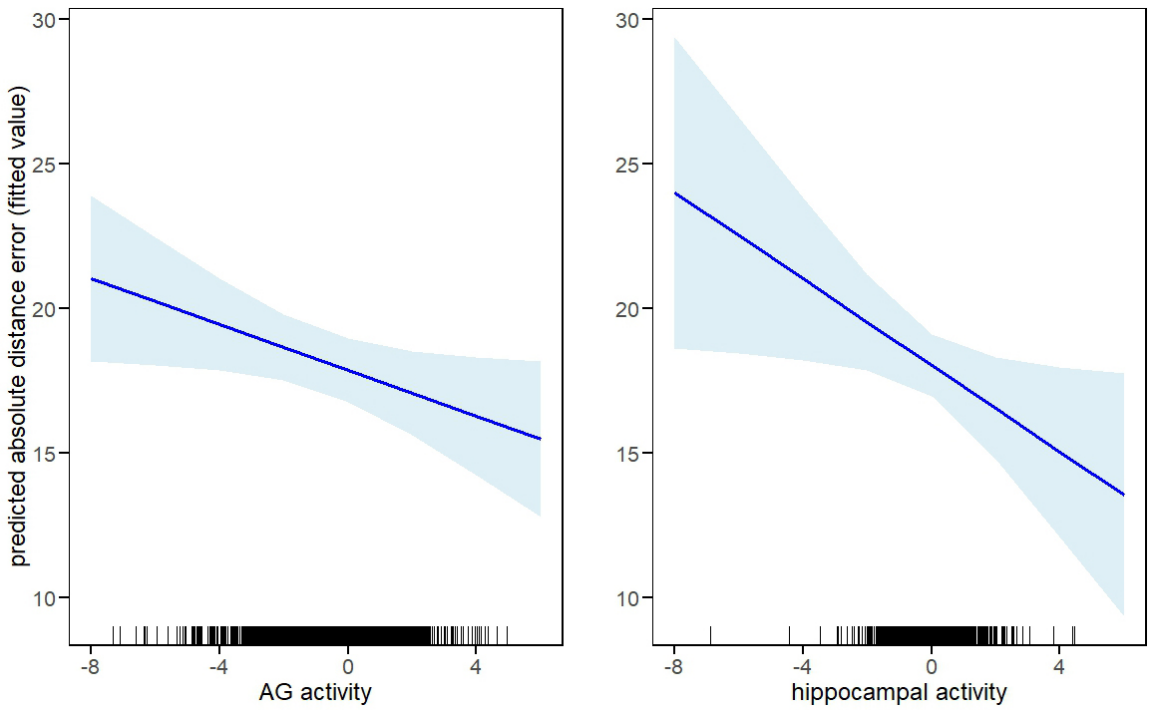
Plots depicting the relationships between trial-wise neural activity in the AG and the hippocampus and the model fitted values of memory precision for location hits, as derived from the LME model in which the two regions served as joint predictors of precision. The shaded areas reflect the 95% confidence intervals. The results were unaltered by the exclusion of trials on which fMRI BOLD parameter estimates were greater than 3 SDs from the mean for either the left AG or the hippocampus.

As described in section 2.6.5, we employed LME models to examine the relationships between trial-wise neural activity in the AG and hippocampus and the memory precision of location hits. In these models, we employed single-trial parameter estimates extracted from the AG ROI and the functionally defined hippocampal cluster (see preceding section) as predictors of the absolute distance error. The results are summarized in Table 6. As is evident from the table, trial-wise BOLD activity in both the AG and hippocampus was predictive of absolute distance error, such that greater activity was associated with lower error (and thus higher precision). Of importance, when they were included in the same regression model, hippocampal and AG parameter estimates independently predicted memory precision (see Fig. 6).

**Table 6.** Results from linear mixed models examining the relationships between memory precision and trial-wise BOLD activity extracted from the combined AG and the hippocampal cluster.

Parameter	B (SE)	df	t	p
AG				
AG	-0.51 (0.18)	2382.58	2.85	0.004
Exp	-3.15 (0.74)	47.43	4.27	<0.001
Hippocampus				
Hippocampus	-0.95 (0.33)	2439.80	2.89	0.004
Exp	-3.24 (0.74)	46.96	4.38	<0.001
AG + Hippocampus				
Hippocampus	-0.74 (0.34)	2442.83	2.19	0.028
AG	-0.40 (0.19)	2401.01	2.13	0.033
Exp	-3.09 (0.74)	47.49	4.19	0.001

Note. In an initial iteration of these model, the neural activity x experiment term was not significant in the models employing either AG or hippocampal activity as predictors (ps > 0.524).

#### 3.2.5 Item-level reinstatement

**Fig. 7.**
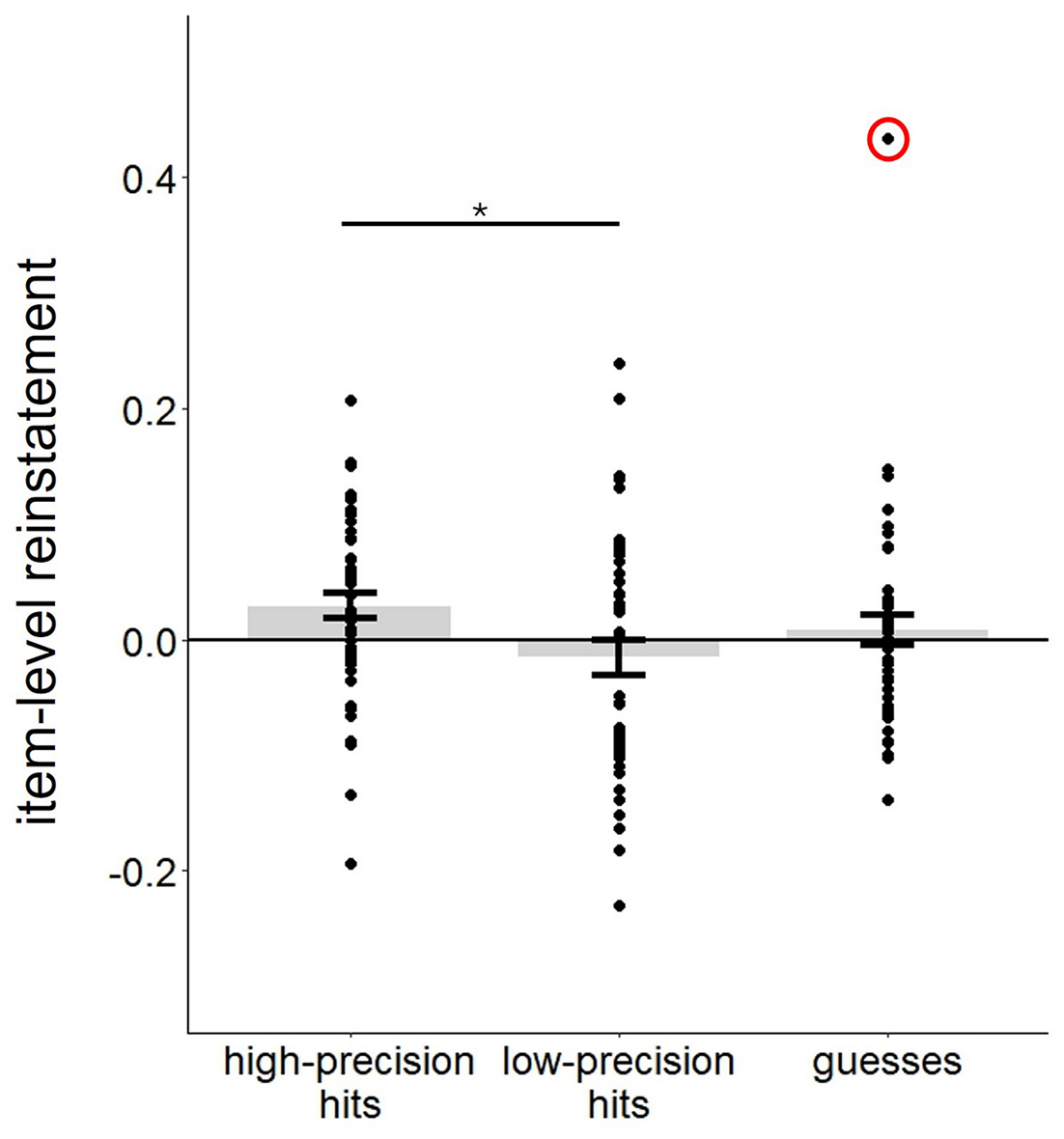
Estimates of item-level reinstatement in the anatomically defined AG. Excluding data from the participant with the outlying data point (circled), the estimate of high-precision hits was greater than both low-precision hits and guesses (ps < 0.016, see main text). *p < 0.05.

Using PSA, we examined whether memory precision was associated with item-level reinstatement, namely the correspondence between item-specific patterns of neural activity elicited during encoding and retrieval. A reliable item-level reinstatement effect was evident in the anatomically defined AG (see section 2) in association with high-precision trials [t(46) = 2.56, p = 0.014, Cohen’s d = 0.37, see Fig. 7]. By contrast, neither low-precision trials nor guesses were associated with above-chance reinstatement (ps > 0.550). No reinstatement effects were identified for any trial type in the hippocampus (ps > 0.652). The 3 (trial type) x 2 (experiment) mixed ANOVA for the AG ROI revealed a significant main effect of experiment, indicative of greater mean pattern similarity in experiment 1 (M = 0.02) than experiment 2 (M = -0.01). Although the main effect of trial type (p = 0.067) and the trial type x experiment interaction were both non-significant (p = 0.399), planned pairwise t-tests revealed a greater reinstatement effect for high- than for low-precision trials [t(46) = 2.42, p = 0.020, Cohen’s d = 0.35; for the comparison between high-precision and guess trials, p = 0.289, see Fig. 7].

We repeated the ANOVA after excluding the data from the participant with the outlying data point (> 3 SDs from the group mean) indicated in Figure 7. The ANOVA revealed significant main effects of trial type [F(1.85, 81.52) = 5.01, p = 0.010, partial η^2^ = 0.102] and experiment [F(1, 44) = 7.29, p = 0.010, partial η^2^ = 0.142], while the trial type x experiment interaction was not significant (p = 0.553). Pairwise contrasts indicated that the reinstatement effect associated with high precision hits was now greater than both low-precision and guess trials [respectively: t(45) = 2.82, p = 0.007, Cohen’s d = 0.42; t(45) = 2.54, p = 0.015, Cohen’s d = 0.37]. At the request of a reviewer, we also conducted linear mixed-effects analyses to examine reinstatement effects associated with the three trial types on a trial-wise basis. Consistent with the findings reported above, these analyses revealed a robust reinstatement effect in the high-precision trials only (see Supplementary Materials).

## 4 Discussion

The present study examined the neural correlates of the retrieval of object location memories, distinguishing between retrieval success and memory precision. The study extended that of Hou et al. (2025; also referred to as experiment 1) by employing an experimental design that reduced the temporal overlap that previously existed between mnemonic and visuomotor processes. With the modified design, several of the cortical effects differing according to the type of memory judgment that were reported in the prior study were no longer detectable. In analyses that extended across the prior and present experiment, we identified significant precision effects in the left AG and the hippocampus. Additionally, linear mixed-effects analyses revealed that neural activity in these two regions independently predicted memory precision on a trial-wise basis. Moreover, in the left AG, across-voxel patterns of neural activity elicited in the study and test phases demonstrated reliable overlap for objects whose locations were remembered with high precision. These findings are discussed in more detail below.

An important goal of the current study was to elucidate the functional significance of the whole-brain trial type effects reported by Hou et al. (2025). As was noted in the Introduction, Hou et al. identified significant differences in the neural activity associated with location hits, guesses, and correct rejections across a variety of cortical and subcortical regions. However, because the test design incorporated a temporal overlap between location recall and the corresponding location judgment, the functional significance of these differences was ambiguous. Here, we modified the design employed by Hou et al. (2025) by temporally separating the memory recall phase from the subsequent memory judgment, thereby removing the aforementioned overlap. With this modification, whole-brain trial type effects were no longer detectable in the left DLPFC or the cerebellum. Thus, consistent with prior evidence implicating these regions in visuomotor function (for reviews, see Schall, 2015; Tzvi et al., 2022), these findings suggest that the judgment effects in these regions reported in Hou et al. (2025) were associated with the varying visuomotor demands associated with cursor placement across the different classes of trial [trial type effects were also absent in the present experiment in the bilateral AG. We have no ready explanation why these effects, which took the form of enhanced activity for correct rejections (‘novelty effects’) should have been impacted by the change in experimental design]. By contrast, trial type effects remained significant in the present experiment in the dorsal parietal and premotor cortex, taking the form, as in Hou et al. (2025), of greater activity for location hits and guesses relative to correct rejections (albeit the effects in bilateral DPC did not survive correction for multiple comparisons). We conjecture that these across-experiment effects reflect differences either in oculomotor activity or motor planning operations associated with the different classes of trial.

Given that we did not identify any reliable between-experiment differences in respect to the neural correlates of retrieval precision, we henceforth focus on the findings from the analyses of the combined dataset. Consistent with prior findings (Cooper et al., 2017; Korkki et al., 2023; Richter et al., 2016), we identified a memory precision effect in the left AG that took the form of greater BOLD activity for locations remembered with high relative to low precision. However, as was also reported for the analysis of experiment 1 alone (Hou et al., 2025; see also Cooper et al., 2017; Richter et al., 2016), we were unable to identify evidence of a retrieval success effect (low precision hits > guesses) in the left AG. On their face, these null findings appear to be inconsistent with the proposal that this region is sensitive to memory precision: precision obviously is greater for low-precision judgments than for those made in the absence of any diagnostic information at all (guesses). As discussed previously (Hou et al., 2025), these null results might, however, reflect the fact that, while bereft of information diagnostic of location information, trials associated with ‘guesses’ were associated with recollection of non-diagnostic information. That is, while location hit trials were a consequence of the successful encoding of an item–location association, guess trials arose when participants devoted attention to a feature of the study event other than the study object’s location. Since memory content was not assessed for any feature apart from location in the present experiments, the validity of this proposal cannot be assessed based on current data. Future studies incorporating additional memory measures into the positional response accuracy paradigm would help to test this possibility.

In addition to the findings for the left AG, we also identified a hippocampal cluster that exhibited a significant memory precision effect (we caution, however, that these findings were derived from an unplanned exploratory analysis, and should be treated as provisional). As was reviewed in the Introduction, out of the four published fMRI studies that examined the neural correlates of precision, only Korkki et al. (2023) reported a significant precision effect in the hippocampus. This mixed evidence stands in contrast to lesion evidence indicating that patients with medial temporal lobe lesions that extend into the hippocampus demonstrate lower memory precision than patients with exclusively extrahippocampal lesions (see section 1). The present findings are consistent with these prior results (and those of Korkki et al. 2023) in suggesting that the hippocampus plays a specific role in high fidelity recollection (see also Ekstrom & Yonelinas, 2020).

Of importance, our findings go beyond prior reports in demonstrating that fMRI BOLD activity in the left AG and hippocampus explains unique components of across-trial variance in memory precision. These regions have long been held to play different roles in memory retrieval. The hippocampus is considered crucial for the encoding of item-context associations and for supporting the retrieval-related reinstatement of neocortical representations of these associations (e.g. Rugg et al., 2015). By contrast, among other possible functions, the left AG has been proposed to support a multi-modal ‘episodic buffer’ that makes episodic content available to executive control processes (King et al., 2015, Rugg & King, 2018; Vilberg & Rugg, 2009, see also Humphreys et al., 2021). The present results suggest that, in cognitively healthy young adults, the contribution of the left AG to memory precision adds to that of the hippocampus. Of course, this does not mean that the AG plays an independent role in precision-based memory judgments. Indeed, based on the available evidence, a likely scenario is that the mnemonic information on which the AG operates depends upon hippocampally-mediated retrieval processing (Rugg et al., 2015; Staresina & Wimber, 2019; Vilberg & Rugg, 2012).

In addition to the univariate precision effects discussed above, the left AG also demonstrated an item-related reinstatement effect that was uniquely associated with high precision judgments. Consistent with our results, prior studies employing multivoxel analysis approaches have also reported AG reinstatement effects for study events that were later retrieved with high fidelity (Kuhl & Chun, 2014; Lee et al., 2019). These findings raise the possibility that the AG plays a role in the reactivation or reinstatement of fine-grained representations of prior events. It remains to be determined whether these effects reflect the fidelity with which the events are represented at the time of encoding, the fidelity of the retrieved episodic representations, or some combination of the two (see Hill et al., 2021, for evidence favoring the first of these possibilities). In contrast to these findings for the left AG, we were unable to identify an analogous reinstatement effect in the hippocampus. This null finding should be treated with caution, however, given the relatively coarse spatial resolution (2.5 mm isotropic voxels) of the fMRI methods employed in both the prior and present experiment.

## 5 Limitations

There are several limitations to the current study. First, we only assessed location memory, leaving it unresolved whether the findings, particularly those related to item reinstatement, generalize to memory for other features. Second, we were unable to assess the potential influence of memory for nondiagnostic information on the BOLD responses elicited by studied test items. And third, as already alluded to, the spatial resolution of the fMRI data in the present study was too low to identify memory effects at the level of hippocampal subfields. These limitations are all addressable in future research.

## 6 Conclusion

Contrasts of the whole-brain results from our prior (Hou et al., 2025) and the present study revealed that several, but not all of those results were likely a consequence of employing an experimental design in which mnemonic and visuomotor processing demands overlapped temporally. The functional significance of the results common to the two studies remains to be established. Additionally, analysis of the combined datasets revealed that, as indexed by fMRI BOLD signals, neural responses in the left angular gyrus and the hippocampus explain independent components of trial-wise variance in memory precision. These findings suggest that these regions play non-redundant roles in supporting the retrieval and behavioral expression of high-fidelity episodic memories.

## Supplementary Material

Supplementary Material

## Data Availability

The data generated by this study are undergoing additional analyses. The data that support the findings of this study are available from the authors on request subsequent to a formal data-sharing agreement. The software, functions, formulas, and syntax of the model used to generate the results are specified in section 2.
