# Supplementary material for: fMRI BOLD signals in the left angular gyrus and hippocampus are associated with memory precision
Source: Imaging Neurosci (Camb). 2025 Nov 10;3:IMAG.a.977. doi: 10.1162/IMAG.a.977 (PMC12603656; doi:10.1162/IMAG.a.977)
Supplement: Supplementary Material [file IMAG.a.977_supp.pdf]

fMRI BOLD signals in the left angular gyrus and hippocampus are associated with memory precision

Mingzhu Hou, Paul F. Hill, Luke R. Pezanko, Ayse N. Z. Aktas, Arne D. Ekstrom and Michael D. Rugg

Supplementary Materials

1. Results from analyses with parametric modulation
2. Memory effects in the anatomically defined left AG
3. Relationships between neural activity and trial-wise memory precision in location hits and guess trials
4. Trial-wise analyses on item-level reinstatement effect

1. Results from analyses with parametric modulation

As we mentioned in section 2.6.1, we conducted a whole-brain analysis with parametric modulation. In the subject-wise analysis, location hits, guesses and CRs were modeled as events of interest. In addition, trial-wise raw distance error of location hits was modeled as a parametric modulator to estimate the degree to which neural activity varied with precision. We extracted parameter estimates of location hits, guesses and CRs and the parametric modulator from the combined AG ROI and the anatomically defined hippocampus. Estimates from the left and right hippocampus were averaged before statistical analyses. For the precision effect, parameter estimates of the parametric modulator from each ROI were subject to a one-sample t-test (initial two-sample t-tests did not reveal a difference between experiments, $ps > 0.600$). Beta estimates of the event of interest were subject to a 3 (trial type: location hits, guesses, CRs) x 2 (experiment) x 2 (region) ANOVA.

A significant precision effect was identified in the combined AG ROI [$t(46) = 3.04$, $p = 0.004$, Cohen's $d = 0.44$]. By contrast, no such effect was observed in the hippocampus ($p = 0.243$). Results of the ANOVA were shown in Supplementary Table 1. As is evident from the table, both main effects of region and experiment attained significance, which were further qualified by an ROI x experiment interaction. Pairwise t-tests following up the interaction revealed that experiment 1 was associated with weaker activity than experiment 2 in the AG ROI (respectively for experiment 1 and 2: $M = -0.71$, $M = -0.37$) but not the hippocampal ROI (respectively for experiment 1 and 2: $M = -0.13$, $M = -0.08$). Moreover, there was a significant effect of trial type, along with a marginally significant trial type x experiment interaction. Follow-up analyses revealed that in experiment 1, CRs were associated with greater activity than both location hits and guesses (respectively, $M = -0.28$, -0.48 and -0.50, $ts > 3.62$, $ps = 0.001$). There was no significant difference between location hits and guesses ($p = 0.632$). No significant difference was observed among trial types in experiment 2 ($ps > 0.607$).

Therefore, consistent with the results from the categorical analyses reported in the main text, we identified a significant memory precision effect in the AG but not the hippocampal ROI. Neither ROI showed a significant retrieval success effect.

Supplementary Table 1. Results of ANOVAs comparing location hits, guesses and CR trials in the AG and hippocampal ROIs.

	Results
Trial type	**$F(1.81, 81.34) = 5.54$, $p = 0.007$, partial $\eta 2 = 0.110$**

Region	**$F(1, 45) = 91.91$, $p < 0.001$, partial $\eta 2 = 0.674$**
Experiment	**$F(1, 45) = 8.80$, $p = 0.005$, partial $\eta 2 = 0.164$**
Trial type x Region	$F(1.84, 82.85) = 2.39$, $p = 0.102$, partial $\eta 2 = 0.050$
Trial type x Experiment	$F(1.81, 81.34) = 3.22$, $p = 0.050$, partial $\eta 2 = 0.067$
Region x Experiment	**$F(1, 45) = 10.05$, $p = 0.003$, partial $\eta 2 = 0.183$**
Trial type x Region x Experiment	$F(1.84, 82.85) = 0.97$, $p = 0.378$, partial $\eta 2 = 0.021$

2. Memory effects in the anatomically defined left AG

To examine whether the results from the combined AG ROI could generalize to the anatomically defined AG (PGap), we repeated the categorical and linear mixed effects analyses employing parameter estimates extracted from PGap. For the categorical analyses, a 3 (trial type: high-precision hits, low-precision hits, guesses) x 2 (experiment) ANOVA revealed a significant main effect of trial type [$F(1.92, 86.32) = 4.74$, $p = 0.012$, partial $\eta 2 = 0.095$]. The effects of experiment ($p = 0.061$) and trial type x experiment interaction ($p = 0.661$) did not reach significance. Pairwise t-tests following up the trial type effect showed greater activity for the high-precision hits than both low-precision hits [$t(46) = 2.80$, $p = 0.007$, Cohen's $d = 0.41$] and guesses [$t(46) = 2.30$, $p = 0.026$, Cohen's $d = 0.34$]. The activity did not differ significantly between low-precision hits and CRs ($p = 0.354$).

We also constructed a linear mixed effects model to examine whether PGap activity was associated with trial-wise memory precision for location hits. As is evident from Supplementary Table 2, PGap activity significantly predicted memory precision. Therefore, the results based on PGap are highly consistent with those from the combined AG ROI reported in the main text.

Supplementary Table 2. Results from linear mixed model examining the relationship between memory precision and trial-wise BOLD activity from the PGap.

Parameter	B(SE)	df	t	p
PGap				
PGap	**-0.47 (0.20)**	**2424.26**	**2.32**	**0.020**
Exp	**-3.27 (0.73)**	**46.71**	**4.45**	**< 0.001**

Note. The neural activity x experiment interaction did not attain significance ($p = 0.763$) in the initial model examining the relationship between PGap activity and memory precision.

3. Relationships between neural activity and trial-wise memory precision in location hits and guess trials

We expanded the original lme analyses described in section 2.6.5 to examine the relationships between neural activity and trial-wise precision for both location hits and guesses. In the models each focusing on the AG and hippocampal activity, experiment, neural activity and trial type (location hits and guesses as 2 levels), as well as the neural activity x trial type interaction were employed as fixed effects. The activity x experiment x trial type interaction was not significant in the initial models and therefore removed from the analyses. Participants were modeled as a random intercept.

We used the anova() function in the lme4 package to evaluate the contribution of each fixed effect to trial-wise memory precision and presented the results in Supplementary Table 3. As is evident from the table, AG activity significantly interacted with trial type. The neural activity x trial type interaction approached significance in the hippocampus. We further examined the relationship between neural activity and trial-wise absolute distance error selectively for the guess trials. As is shown in Supplementary Table 4, activity in neither ROI significantly predicted precision of these trials.

Supplementary Table 3. Results from linear mixed-effects models examining the relationships between neural activity, trial type, and trial-wise memory precision. Fixed effects were assessed using the anova() function.

Parameter	Df	F	p
AG			
AG	(1, 3214.0)	0.68	0.409
Trial type	**(1, 3261.1)**	**9117.47**	**< 0.001**
Exp	**(1, 46.3)**	**5.84**	**0.020**
AG x Trial type	**(1, 3682.7)**	**6.58**	**0.010**
Hippocampus			
Hippocampus	(1, 3623.7)	<0.01	0.973
Trial type	**(1, 3139.1)**	**10439.4**	**< 0.001**
Exp	**(1, 44.9)**	**5.56**	**0.023**
Hippocampus x Trial type	(1, 3688.6)	2.96	0.085

Supplementary Table 4. Results from linear mixed models examining the relationships between memory precision for guess trials and BOLD activity extracted from the combined AG and the hippocampal cluster.

Parameter	B(SE)	df	t	p
AG				
AG	0.99 (0.84)	1243	1.18	0.238
Exp	-1.00 (2.29)	1243	0.44	0.664
Hippocampus				
Hippocampus	0.92 (1.46)	1243	0.63	0.526
Exp	-0.64 (2.27)	1243	0.28	0.777

4. Trial-wise analyses on item-level reinstatement effect

We constructed a linear mixed effects model to examine the trial-wise item-level reinstatement effects of high-precision, low-precision hits and guesses. The model involving AG included experiment, trial type (3 levels: high-precision hits, low-precision hits, guesses), and their interaction as the fixed effects predicting the trial-wise item-level reinstatement effect. Participants were modeled as a random intercept. Given that the trial type x experiment interaction was not significant in the initial model, we repeated the analysis after removing this term. The results are presented in Supplementary Table 5. As is shown in the table, neither the effect of trial type nor experiment attained significance.

We further tested whether each trial type exhibited a robust item-level reinstatement effect. Estimated marginal means from the previously described model were contrasted against zero. While the high-precision trials showed a reliable reinstatement effect (emmean = 0.023, p = 0.029), the effect of low-precision and guesses trials did not differ significantly from 0 (emmeans < 0.015, ps > 0.213). Analogous analyses conducted with the anatomically defined hippocampus did not reveal a significant main effect of trial type or robust reinstatement effects for any trial type (ps > 0.350).

Supplementary Table 5. Results from the linear mixed-effects model examining AG item-related reinstatement effect among different trial types. Fixed effects were assessed using the anova() function.

Parameter	Df	F	p

AG item-level reinstatement			
Trial type	(2, 2922)	1.04	0.354
Exp	(1, 2922)	1.35	0.245
